# A polybasic motif in ErbB3-binding protein 1 (EBP1) has key functions in nucleolar localization and polyphosphoinositide interaction

**DOI:** 10.1042/BCJ20160274

**Published:** 2016-07-12

**Authors:** Thomas Karlsson, Altanchimeg Altankhuyag, Olena Dobrovolska, Diana C. Turcu, Aurélia E. Lewis

**Affiliations:** * NucReg Research Program, Department of Molecular Biology, University of Bergen, 5008 Bergen, Norway

**Keywords:** EBP1, interaction, nucleolus, p110β, PI3K, PIP3

## Abstract

Polyphosphoinositides (PPIns) are present in the nucleus where they participate in crucial nuclear processes, such as chromatin remodelling, transcription and mRNA processing. In a previous interactomics study, aimed to gain further insight into nuclear PPIns functions, we identified ErbB3 binding protein 1 (EBP1) as a potential nuclear PPIn-binding protein in a lipid pull-down screen. EBP1 is a ubiquitous and conserved protein, located in both the cytoplasm and nucleolus, and associated with cell proliferation and survival. In the present study, we show that EBP1 binds directly to several PPIns via two distinct PPIn-binding sites consisting of clusters of lysine residues and positioned at the N- and C-termini of the protein. Using interaction mutants, we show that the C-terminal PPIn-binding motif contributes the most to the localization of EBP1 in the nucleolus. Importantly, a K372N point mutation, located within the C-terminal motif and found in endometrial tumours, is sufficient to alter the nucleolar targeting of EBP1. Our study reveals also the presence of the class I phosphoinositide 3-kinase (PI3K) catalytic subunit p110β and its product PtdIns(3,4,5)*P*_3_ together with EBP1 in the nucleolus. Using NMR, we further demonstrate an association between EBP1 and PtdIns(3,4,5)*P*_3_ via both electrostatic and hydrophobic interactions. Taken together, these results show that EBP1 interacts directly with PPIns and associate with PtdIns(3,4,5)*P*_3_ in the nucleolus. The presence of p110β and PtdIns(3,4,5)*P*_3_ in the nucleolus indicates their potential role in regulating nucleolar processes, at least via EBP1.

## Abstract (precis)

We reveal the identification of a polybasic motif necessary for polyphosphoinositide interaction and nucleolar targeting of ErbB3 binding protein 1 (EBP1). EBP1 interacts directly with phosphatidylinositol(3,4,5)-triphosphate and their association is detected in the nucleolus, implying regulatory roles of nucleolar processes.

## INTRODUCTION

Phospholipids are well known to play fundamental roles not only as structural components of membranes but also in signal transduction pathways initiated at the plasma membrane. However they have also emerged as essential components of the nucleus not only in the nuclear envelope but also within nuclei, in the nuclear matrix and in association with the chromatin [1,2]. This endonuclear pool of phospholipids represents approximately 6–10% of the total cell composition of phospholipids [3]. Polyphosphoinositides (PPIn nomenclature from [4]), which consist of seven phosphorylated derivatives of phosphatidylinositol (PtdIns), are also present within the nucleus together with the enzymes that regulate their interconversion [5–8]. With the exception of PtdIns(3,4)*P*_2_ and PtdIns(3,5)*P*_2_, the other five PPIns have been detected within the nucleus deprived of their nuclear envelope, by radiolabelling and mass assays [9–14], electron microscopy or by immunofluorescence using specific PPIn probes or antibodies [15–20]. PPIns regulate nuclear processes, such as protein–chromatin association, transcription, pre mRNA processing, splicing and export as well as cell cycle progression [21–25], by interacting with proteins containing PPIn-binding domains [26] or polybasic regions (PBR)/K/R motifs [24,27]. Mono-phosphorylated PPIns were shown to interact with Pf1 (plant homeodomain zinc finger 1) [28], SAP30L (Sin3A-associated protein 30 like) [29] and ING2 (inhibitor of growth protein 2) [30,31], proteins known to bind the co-repressor Sin3A. PPIns-interaction was further shown to regulate SAP30L and ING2 association to chromatin [13,29,30]. BRG1 (Brahma related gene 1), a component of the chromatin remodelling BAF complex, binds to PtdIns(4,5)*P*_2_ and this interaction regulates E-actin association to the complex [32,33]. Nuclear PPIn functions include also pre-mRNA processing such as splicing and polyadenylation as well as mRNA export to the cytoplasm [17–19,34,35]. A pool of PtdIns(4,5)*P*_2_ present in nuclear speckles binds to and regulates the activity of the poly(A) polymerase Star-PAP [nuclear speckle targeted PIPKIα regulated-poly(A) polymerase] [36]. Furthermore, ALY (alias THO complex subunit 4) binds to both PtdIns(4,5)*P*_2_ and PtdIns(3,4,5)*P*_3_, an interaction essential for its localization to nuclear speckles and mRNA export [35,37]. Regarding transcriptional regulation, several studies have correlated gene expression to the interaction of the nuclear receptors SF-1 (steroidogenic factor-1) and LRH-1 (liver receptor homologue-1) with PtdIns(4,5)*P*_2_ and PtdIns(3,4,5)*P*_3_ in the ligand binding pocket [38–41] and the basal transcription factor TAF3 (TATA box binding protein-associated factor 3) to several PPIns via a PBR [42]. PtdIns(4,5)*P*_2_ was also shown to bind to BASP1 (brain acid soluble protein 1) and this interaction promotes a co-repressive function by recruiting histone deacetylases [43]. Other data correlate changes in the levels of nuclear PPIns to cell cycle progression [12,44,45] or apoptosis via an interaction between nucleophosmin (NPM) and PtdIns(3,4,5)*P*_3_ [46].

These studies clearly show the importance of PPIns in the nucleus but they represent only a few examples of nuclear PPIn-binding proteins. To further define the global significance of nuclear PPIns, we have previously established a quantitative proteomic method to identify nuclear PPIn-interacting proteins [24]. Using SILAC metabolic labelling, lipid pull-down and MS, we identified 28 nuclear proteins as potential PtdIns(4,5)*P*_2_-binding proteins, among which ErbB3 binding protein 1 (EBP1), alias proliferation-associated protein 2G4 (PA2G4). EBP1 is conserved through evolution and ubiquitously expressed [47–49]. Human EBP1 was originally identified as a binding partner of the tyrosine kinase receptor ErbB3 [50] and as the homologue of murine p38-2AG4 [47]. *PA2G4* encodes two splice variants, a long and predominant form p48, and a shorter and minor form p42 (homologue to p38-2G4) which differs in its N-terminus by the lack of the first 54 amino acids [51]. The two isoforms have distinct sub-cellular localizations, p48-EBP1 is present in the cytoplasm, nucleus as well as the nucleolus [52,53], whereas p42-EBP1 is restricted to the cytoplasm, where it is targeted for degradation via its ubiquitination [51,54]. p48-EBP1 has the ability to translocate from the cytoplasm to the nucleus upon the activation of ErbB3 [50] or upon high cell density in oral squamous carcinoma cells [55].

In the present study, we show that EBP1 binds directly to several PPIn species via two different PPIn interaction sites consisting of lysine-rich PBRs located in the two termini of the protein. The two PBRs have a different PPIn-interaction profile and contribute to EBP1 nucleolar localization, albeit differently. EBP1 interacts particularly with PtdIns(3,4,5)*P*_3_ via its C-terminal PBR and this association is localized in the nucleolus. The C-terminal PBR is mutated in endometrial cancer and we showed that this partially prevents PtdIns(3,4,5)*P*_3_-interaction as well as its nucleolar localization. These data demonstrate that the EBP1 PBRs have a dual function as a PPIn interaction motif and nucleolar localization signal, and imply that the regulation of EBP1-mediated nucleolar processes is potentially regulated by PtdIns(3,4,5)*P*_3_.

## MATERIALS AND METHODS

### Plasmids, cloning and site-directed mutagenesis

The phospholipase Cδ1 pleckstrin homology domain (PLCδ1-PH) cloned in pGEX-4T was obtained from Dr A.Z. Gray (University of Dundee, UK) and the general receptor for phosphoinositides-1 PH domain (GST-GRP1-PH) cloned in pGEX-4T3 was from Dr J. Hastie (MRC, University of Dundee, UK). pGEX-4T2-hEBP1 and pEGFP-C2-hEBP1 were from M. Squatrito [53]. The N- and C-terminal EBP1 fragments were amplified by PCR from pGEX-4T2-hEBP1 using primers flanked by EcoRI restriction sites and cloned into pGEX-4T1. All mutants were generated by QuickChange site-directed mutagenesis (Agilent Technologies) according to the manufacturer's instructions and verified by sequencing using ABI Prism BigDye Terminator version 3.1 cycle sequencing kit (Applied Biosystems). GRP1-PH was amplified by PCR from pGEX-4T3-GRP1-PH using primers flanked by BglII and SalI restriction sites and subcloned into pEGFP-C2-NLS. All primers are listed in Supplementary Table S1.

### GST-tagged protein expression and purification

GST-PLCδ1-PH and GST-GRP1-PH were expressed and purified as described previously [24]. GST-EBP1, full length (FL), N- and C-terminal fragments, WT and mutants were transformed into *Escherichia coli* BL21-RIL DE3 and bacterial cultures were grown at 37°C and further induced with 0.5 mM isopropyl-E-D-thiogalactopyranoside for 3 h at 37°C. Bacterial pellets were resuspended in 50 mM Tris pH 7.5, 2 mM EDTA, 1 mM DTT and 1x Sigma protease inhibitor cocktail, sonicated three times for 30 s at 4°C and centrifuged at 4400 ***g*** for 10 min at 4°C. GST-tagged proteins were purified with glutathione-agarose 4B beads from an overnight pull down, eluted with 50 mM Tris pH 8.0, 100 mM NaCl, 0.5 mM DTT and 10 mM reduced glutathione, and analysed by SDS/PAGE and Coomassie staining for purity. For NMR studies, *E.coli* were grown in M9 minimal medium, supplemented with 6 g/l Na_2_HPO_4_, 3 g/l KH_2_PO_4_, 0.5 g/l NaCl, 0.25 g/l MgSO_4_, 1 g/l 98%-enriched (^15^NH_4_)_2_SO_4_ and/or 4 g/l ^13^C6-glucose to produce ^15^N- and/or ^13^C-uniformly labelled GST-C-terminal domain of EBP1. Further protein expression and purification was performed using the same protocol as described above.

### Lipid overlay assays

Lipid overlay assay was carried out using PIP Strips™ (Echelon Biosciences) spotted with 100 pmol of each of the 7 PPIns in addition to other phospholipids, and PIP Arrays™ spotted with 1.56–100 pmol of PtdIns or each of the 7 PPIns. PIP strips™ and arrays were incubated with blocking buffer (3% fatty acid-free BSA (Sigma A6003) in TBS-T (50 mM Tris pH 7.5, 150 mM NaCl, 0.1% Tween-20) for 1 h at room temperature. PIP Strips™ were incubated with 1.5 μg/ml GST-tagged protein or dialysed neomycin extracts in the same buffer overnight at 4°C. Detection of GST-tagged proteins and EBP1 (from neomycin extracts) was performed with an anti-GST-HRP conjugated antibody (Abcam, ab3416, 1:50000) and an anti-EBP1 antibody (M. Squatrito, 1:800) respectively, both diluted in blocking buffer. PIP arrays™ were incubated with anti-PtdIns(3,4,5)*P*_3_ (Echelon, #Z-P345b, 1:10000) followed by anti-mouse IgG-HRP (1:10000), both diluted in blocking buffer made in PBS-T (137 mM NaCl, 2.68 mM KCl, 8 mM Na_2_HPO_4_, 1.8 mM KH_2_PO_4_, 0.1% Tween-20). Six washes of 5 min each with TBS-T or PBS-T were performed after incubations with protein and antibody. The protein–lipid interactions were visualized using a west pico or femto chemiluminescent substrate and a Bio-Rad ChemiDoc™ XRS+Imaging System from Bio-Rad and the ImageLab™ Software Version 3.0.

Relative binding of EBP1 to the phospholipids was quantified by densitometry using ImageJ software (http://rsb.info.nih.gov/ij). The data were normalized to background signals for each blot.

### NMR spectroscopy

The NMR sample contained 0.14 mM uniformly ^15^N- and/or ^13^C-labelled GST-CTD-EBP1 in 50 mM Tris buffer S+5.5 containing 100 mM NaCl, 0.5 mM 2-mercaptoethanol and 90% H_2_O/10% D_2_O. NMR spectra were acquired at 298 K on a Bruker Avance spectrometer operating at proton frequency of 600.13 MHz using the acquisition parameters provided in Supplementary Table S2. The spectrometer was equipped with a TCI 5-mm triple resonance cryo-probe with pulse field gradients along the *z*-axis. Spectra were recorded and processed in TopSpin 2.1 (Bruker Biospin). ^1^H, ^13^C and ^15^N backbone resonance assignments for the protein were determined using CARA (Computer Aided Resonance Assignment) version 1.8.4.2 [56]. Secondary structure propensities (SSP) were calculated with the Cα and Cβ chemical shifts as input into the SSP algorithm [57]. To monitor protein-lipid binding ^1^H–^15^N heteronuclear single quantum coherence (HSQC) spectra acquired in the absence and presence of 0.08 mM diC16-PtdIns(3,4,5)*P*_3_ or 0.33 mM diC8-PtdIns(3,4,5)*P*_3_ (Echelon Biosciences) were subsequently compared and analysed. The peak intensities were measured in CARA using peak-fitting algorithm [56]. The robustness of the intensity values was tested by using different fit parameters and the variation in the output did not exceed 2%.

### Cell culture and transfection

MEL cells were cultured with DMEM supplemented with 10% foetal bovine serum (FBS) and antibiotics (50000 units of both penicillin and streptomycin) at 37°C with 5% CO_2_. AU565 cells were cultured under the same conditions but in RPMI-1640 medium. For transfections, cells were plated in six-well plates and transfected with 1–2 μg DNA and XtremeGene 9 (Roche) at 3:1 ratio for 24 h. Treatment with the pan-PI3K inhibitor LY294002 (10 μM) or DMSO (0.2% (v/v)) was performed 4 h post-transfection.

### Nuclear fractionation and neomycin extraction

Nuclei were isolated according to a method by Mukai et al. [58] with some modifications. Cells were washed two times in PBS and once briefly in buffer A (10 mM Hepes pH 7.9, 10 mM KCl, 1.5 mM MgCl_2_, 340 mM sucrose, 10% glycerol). Cells were resuspended in buffer A containing 0.1% Triton X-100, 1 mM DTT, 5 μg/ml leupeptin and 5 μg/ml aprotinin, left to swell for 5 min on ice and centrifuged at 1300 ***g*** for 5 min at 4°C. Nuclei were washed quickly with retention buffer (20 mM Tris pH 7.5, 70 mM NaCl, 20 mM KCl, 5 mM MgCl_2_ and 3 mM CaCl_2_ [59]). Nuclei were incubated twice in retention buffer for 30 min at room temperature, split into two equal fractions and further incubated in the presence or absence of 5 mM neomycin (trisulfate salt, Sigma N6386) for 30 min at room temperature. Samples were centrifuged at 9600 ***g*** for 5 min at 4°C and super-natants were collected. For lipid overlay assays, neomycin supernatants were dialysed twice against 20 mM HEPES pH 7.5, 150 mM NaCl, 5 mM EDTA and 0.1% NP-40.

### SDS/PAGE and Western immunoblotting

Proteins were resolved by SDS/PAGE and transferred to nitrocellulose membranes. Membranes were blocked with 5% non-fat milk, incubated with primary antibodies overnight at 4°C and with secondary antibodies conjugated to HRP for 1 h at room temperature. Protein detection was performed by chemiluminescence using the SuperSignal West Pico Chemiluminescent Substrate (Pierce) and imaged using the Molecular Imager® ChemiDoc™ XR+Imaging System and the ImageLab™ Software Version 3.0 (Bio-Rad).

### Immunostaining and microscopy

AU565 cells were seeded on 12 mm glass coverslips placed in 12-well plates and cultivated for 24 h before fixation. The cells were washed two times in PBS, fixed in 3.7% paraformaldehyde for 10 min at room temperature. Following fixation, cells were washed three times with PBS, permeabilized with 0.25% Triton X-100 in PBS pH 7.2 for 10 min at room temperature and blocked with 5% goat serum in PBS containing 0.05% Triton X-100 for 1 h at room temperature. Cells were incubated overnight at 4°C with anti-EBP1 (abcam ab33613, 1:200, and antibody from M. Squatrito, 1:800, o/n), anti-NPM (Zymed 32-5200, 1:1000, 1 h), anti-nucleolin (Cell Signaling 14574, 1:100, 1 h), anti-p110β (abcam ab151549, 1:50, o/n), anti-PtdIns(3,4,5)*P*_3_ (Echelon, 1:200, o/n) diluted in blocking buffer, followed by incubation with anti-mouse IgG antibody conjugated to Alexa-594 (1:200) or Alexa-488 (1:400) (Life Technologies) diluted in blocking buffer for 1 h at room temperature. Washes were performed with 0.05% PBS-T after incubation with each antibody. The cover slips were mounted in ProLong® Gold Antifade Reagent containing 4′,6-diamidino-2-phenylindole (DAPI) (Life Technologies). Control staining with secondary antibody alone under the same staining and exposure conditions showed no unspecific staining. Images were acquired with a Leica DMI6000B fluorescence microscope using ×40 or ×100 objectives or with a Leica TCS SP5 confocal laser scanning microscope using a 63×/1.4, oil immersion lens. Images were processed with the Leica application suite v4.0 and Adobe Photoshop CS5.

## RESULTS

### EBP1 binds directly to phosphoinositides

**Figure 1:**
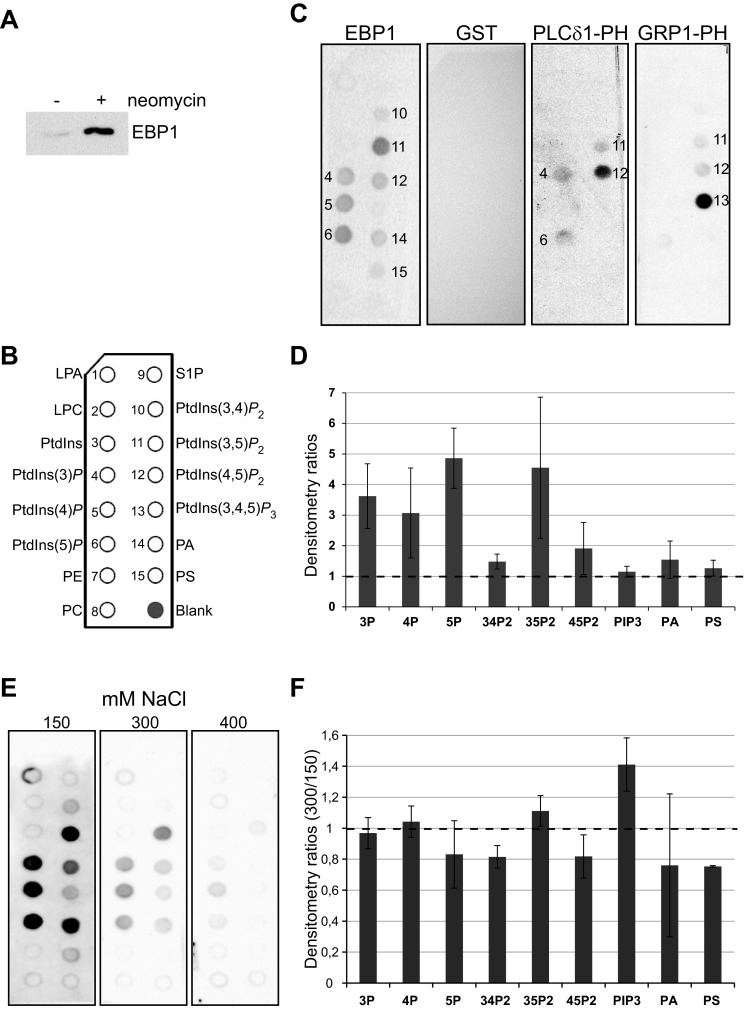
EBP1 binds to phosphoinositides (**A**) MEL nuclei were isolated, washed and incubated in retention buffer without (−) or with (+) 5 mM neomycin for 30 min at RT. Supernatants were analysed by Western immunoblotting. (**B**) PIP strip schematic overview showing the positions of the spotted lipids (www.echelon-inc.com). LPA, lysophosphatidic acid; LPC, lysophosphatidylcholine; PI, phosphatidylinositol; PE, phosphatidylethanolamine; PC, phosphatidylcholine; S1P, sphingosine-1-phosphate; PA, phosphatidic acid; PS, phosphatidylserine. (**C**) PIP strips incubated with recombinant GST-fused proteins (EBP1 FL WT, PLC-δ1-PH and GRP1-PH domains) and detection of protein–lipid interactions using an anti-GST-HRP conjugated antibody. (**D**) Quantification of binding signal from four separate experiments shown as means +S.D. of densitometry ratios related to background signal. (**E**) PIP strips incubated with GST-EBP1 FL in TBS-T containing 150–400 mM NaCl and detection of protein–lipid interactions using an anti-GST-HRP conjugated antibody. (**F**) Quantification of binding signal from two separate experiments shown as means +S.D. of densitometry ratios (300/150) each related to background signals.

We have previously identified EBP1 as a potential PtdIns(4,5)*P*_2_-binding protein by combining PtdIns(4,5)*P*_2_ pull down from neomycin-displaced nuclear proteins and quantitative MS [24]. The peptides which identified EBP1 are shown in Supplementary Figure S1 and include a peptide which only matches the long p48 variant. We first validated that p48-EBP1 could be displaced by neomycin from the nucleus by Western immunoblotting analyses (Figure 1A). We next assessed EBP1 as a direct PPIn-interacting protein by performing lipid overlay assays on phospholipid-immobilized strips (Figure 1B) using recombinant p48-GST-EBP1. As shown in Figure 1(C), GST-EBP1 bound to several PPIn species including the three mono-phosphorylated PPIns and PtdIns(3,5)*P*_2_. Binding to PtdIns(4,5)*P*_2_, PtdIns(3,4)*P*_2_, phosphatidic acid (PA) and phosphatidylserine (PS) is weaker and not always detected (Figures 1C and 1D). GST showed no interaction by itself. Control binding experiments for PtdIns(4,5)*P*_2_ and PtdIns(3,4,5)*P*_3_ were also performed using specific probes, i.e. GST-fused PH domains of PLC-δ1 and GRP1 respectively. As shown in Figure 1(C), the PH domain of PLC-δ1 bound strongly to PtdIns(4,5)*P*_2_, whereas very weak binding was observed for PtdIns3*P*, Ptdins5*P* and PtdIns(3,5)*P*_2_. The PH domain of GRP1 interacted strongly with PtdIns(3,4,5)*P*_3_ and very weakly with both PtdIns(4,5)*P*_2_ and PtdIns(3,5)*P*_2_. Considering that EBP1 interacts with most anionic phospholipids, we tested the effect of increasing NaCl concentration on these interactions (Figures 1E and 1F). Doubling the NaCl concentration had overall little effect on interaction but binding to the three mono-phosphorylated PPIns and PtdIns(3,5)*P*_2_ was essentially the same as measured by retention of the protein to the strips, although PA and PS binding was reduced. Adding 400 mM NaCl nearly abolished all interaction detected except for PtdIns4*P* and PtdIns(3,5)*P*_2_. These results suggest that the interactions with PtdIns3*P*, PtdIns5*P* and particularly with PtdIns4*P* and PtdIns(3,5)*P*_2_ have the highest affinity for the FL EBP1 protein.

### EBP1 binds to PPIns via two lysine-rich PBRs

**Figure 2:**
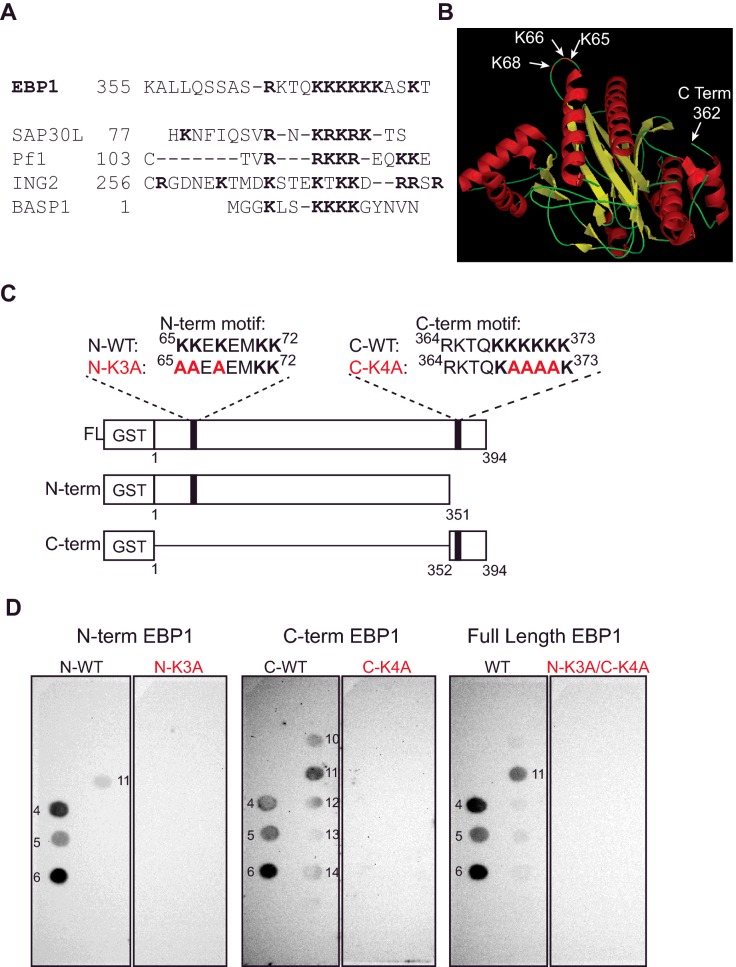
EBP1 harbours two lysine-rich PBRs required for PPIn interaction (**A**) Alignment of the C-terminal lysine-rich PBR of EBP1 with other nuclear PIP-binding proteins. (**B**) 3D structure of EBP1 amino acids 1–361 and location of the N-terminal lysine-rich PBR loop (pdb 2Q8K). (**C**) Representation of the recombinant GST-EBP1 FL, N-terminal (N-term) and C-terminal (C-term) constructs and the approximate locations of the two lysine-rich PBRs as well as their mutants highlighted in red. (**D**) PIP strips were incubated with recombinant GST-EBP1 proteins (FL, N- and C-terminal), WT or the following lysine-rich PBR mutants, N-K3A (K65A–K66A–K68A), C-K4A (K369A–K370A–K371A–K372A) and N-K3A/C-K4A combined mutant. Protein–lipid interactions were detected using an anti-GST-HRP conjugated antibody.

EBP1 does not harbour PPIn-binding modules, such as PH, PX or FYVE domains [60], that could account for the observed interactions. However, stretches of basic amino acids denoted as PBRs [61] or KR-motifs following the sequence K/R-(X*_n_*_=3–7_)-KXKK, have also been implicated in PPIn-binding via electrostatic interactions [62]. Such basic amino acid stretches have since then been identified in several nuclear proteins in complex with PPIn [24] including the nuclear proteins ING2 [30], Pf1 [28], SAP30L [29], and more recently in BASP1 [43], UHRF1 [63] and TAF3 [42] (Figure 2A). EBP1 has a KR-motif in the unstructured C-terminal region (^364^RKTQKKKKKK^373^) [24], named C-term PBR, as well as a reverse KR-motif situated on a protruding loop of the N-terminal part (^65^KKEKEMKK^72^), named N-term PBR (Figures 1A and 1B). These two PBRs are highly conserved, suggesting a functional importance (Supplementary Figure S2). Point mutations of three basic residues out of five to alanines within the PBR of SAP30L (^87^KRKRK^91^ → ^87^KAAAK^91^) led to a significant decrease in binding to monophosphorylated PPIns [29]. In order to investigate if the C-terminal PBR-motif of EBP1 played a similar role in PPIn-binding, four out of six lysines were substituted to alanines in the FL GST-EBP1, resulting in the following quadruple C-term PBR mutant ^368^KAAAAK^373^ (FL-C-K4A). FL-C- K4A was tested for its PPIn-binding properties by lipid overlay assay in parallel with the wild-type (WT) FL GST-EBP1. This mutant did not show significant change in PPIn-binding compared with WT except for a variable decrease in binding to PtdIns(3,5)*P*_2_ (results not shown). A mutant was also produced in the N-terminal PBR motif residing on the protruding loop (Figure 2B), resulting in the FL-N-K2A mutant ^65^AAEKEMKK^72^. Again, this mutant did not show any change in the PPIn-binding pattern compared with WT (results not shown). These results suggested that both motifs may act independently in binding to PPIns. An N-terminal fragment (amino acids 1–351), harbouring the N-term PBR motif and a C-terminal fragment (amino acids 352–394) containing the C-term PBR, were therefore created (Figure 2C and Supplementary Figure S3) and tested in lipid overlay assays (Figure 2D). As shown in Figure 2(D) both fragments retained the ability to bind to PPIns, including the three mono-phosphorylated PPIns and PtdIns(3,5)*P*_2_ for both the N- and C-terminal fragments. The C-terminal fragment was able to bind to the remaining PPIns but with variable intensity. The C-K4A mutant abolished PPIn-binding, when tested in the C-term construct (Figure 2D). The N-K2A mutant showed a great decrease in binding when tested in the N-term construct (results not shown) and binding was completely blocked when an additional lysine at K68 was substituted to alanine (Figure 2D). Finally, when the C-K4A and N-K3A mutants were both introduced in the FL protein, PPIn interaction was completely abolished.

### Nucleolar localization of EBP1 is mediated predominantly by the C-terminal PBR

**Figure 3:**
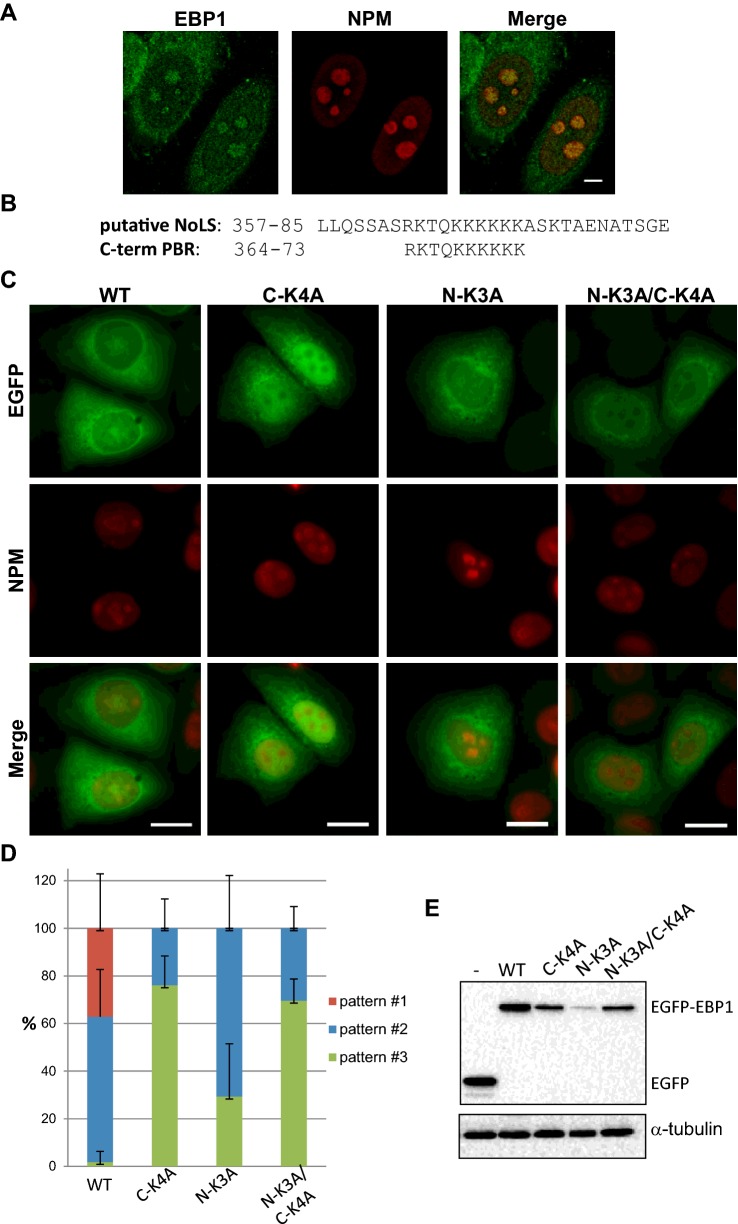
EBP1 localizes to the nucleolus via its C-terminal lysine-rich PBR (**A**) AU565 cells co-stained with anti-EBP1 and anti-NPM antibodies and imaged by confocal microscopy. 5 μmol scale bars. (**B**) Alignment of the putative NoLS sequence and the C-term K-rich PBR of human EBP1. (**C**) AU565 cells transfected with EGFP-C2-EBP1 WT and mutant FL constructs, stained with anti-NPM and imaged by epifluorescence microscopy. Scale bars are all 10 μmol. (**D**) Quantification of the localization patterns of EGFP-EBP1 WT and mutants from at least three different experiments +S.D. (**E**) Western immunoblotting of AU565 cell extracts obtained following transfection with EGFP-C2-EBP1 WT and mutant FL constructs.

EBP1 has previously been shown to localize in the nucleolus of HeLa and NIH-3T3 cells [53] and we confirmed this finding in the breast cancer cell line AU565 cells by co-immunostaining EBP1 with the nucleolar protein NPM. As shown in Figure 3(A), EBP1 was detected both in the cytoplasm, and in punctate foci within NPM-stained nucleoli. The first 48 amino acids in p48-EBP1 were previously shown to be necessary for its nuclear targeting whereas the region spanning amino acids 301–394 was shown to be responsible for its nucleolar localization [53]. Using the nucleolar localization sequence detector (NoD [64]), a nucleolar localization sequence (NoLS) was predicted in EBP1 in amino acids 357–385, which lies within the region previously found to be responsible for nucleolar targeting (Figure 3B and Supplementary Figure S4A). This putative NoLS is well conserved and, interestingly, contains the C-terminal PBR (Figure 3B and Supplementary Figure S4B). We argued therefore that this PPIn-binding motif could contribute to the localization of EBP1 in the nucleolus. AU565 cells were transfected with FL EGFP-tagged WT, C-K4A and N-K3A mutants as well as the N-K3A/C-K4A double mutant and examined by fluorescence microscopy (Figure 3C). In contrast with EGFP alone, which was found in both the cytoplasm and nucleus, EGFP-EBP1 WT and mutants exhibited overall three different patterns of localization (Supplementary Figure S5). The different localization patterns were quantified for WT and each of the mutants (Figure 3D). The first pattern (pattern #1) is characterized by the appearance of EBP1 in the cytoplasm, the peri-nuclear area and the nucleolus, as reported previously [51,53]. This pattern was observed in 37% of cells expressing WT-EBP1 but did not occur in any of the mutants (Figures 3C and 3D). The second pattern (pattern #2) includes either a restricted localization in the cytoplasm with an intense peri-nuclear signal (pattern #2a, Supplementary Figure S5), or a diffuse localization in both cytoplasm and nucleus (pattern #2b, Supplementary Figure S5). Pattern #2 was dominant in cells expressing WT (61%) or the N-K3A mutant (71%) but also occurred in a lower proportion of cells expressing C-K4A (24%) and the double mutant (30%) (Figure 3D). The third pattern (pattern #3), which consists of a cytoplasmic and nucleolar-free localization was mainly observed in cells expressing the C-K4A mutant (76%) and the N-K3A/C-K4A double mutant (70%) and less so for the N-K3A mutant (29%) (Figures 3C and 3D). Some differences were however observed among these mutants for this pattern. The C-K4A mutant was not only excluded from the nucleolus but also strongly retained in the rest of the nucleus (pattern #3c Supplementary Figure S5, Figure 3C). In contrast, the N-K3A mutant and double mutant, which were devoid of nucleolar localization, did not allow the nuclear retention of EBP1 (pattern #3d, Supplementary Figure S5, Figure 3C). The protein levels of all three mutants were lower compared with WT, and the N-K3A mutant had the lowest decrease compared with the other two EBP1 mutants (Figure 3E). These results suggest therefore that the C- and N-term PBRs contribute to the nucleolar localization of EBP1, albeit in a different manner. The C-term PBR has in addition nuclear export properties.

### PtdIns(3,4,5)*P*_3_ is localized in the nucleoplasm and nucleolus

**Figure 4:**
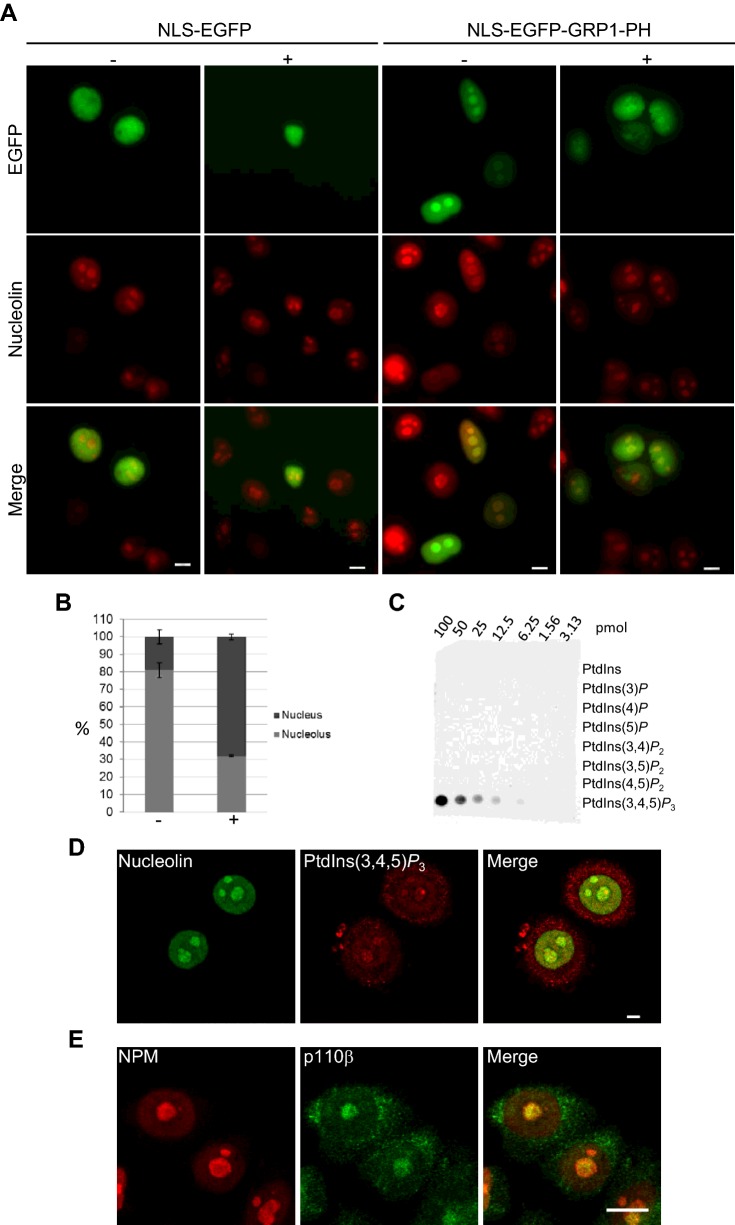
PtdIns(3,4,5)*P*_3_ is localized in nucleoli (**A**) AU565 cells transfected with NLS-EGFP or NLS-EGFP-GRP1-PH and incubated with 10 μmol LY-294002, stained with anti-nucleolin and imaged by epi-fluorescence. (**B**) Quantification of the nucleolar localization of the NLS-EGFP-GRP1-PH from three different experiments +S.D. (**C**) PIP array spotted with 1.56–100 pmol of each of seven PPIn species incubated with an anti-PtdIns(3,4,5)*P*_3_ and an anti-mouse-HRP conjugated antibody. (**D**) Confocal images of AU565 cells co-stained with anti-nucleolin and anti-PtdIns(3,4,5)*P*_3_ antibodies. (**E**) Confocal images of AU565 co-stained with an anti-p110β and anti-NPM antibodies. Scale bars are all 5 μmol.

EBP1-p48 has previously been shown to bind to NPM [65]. Considering that NPM was also shown to be pulled down in a complex with PtdIns(3,4,5)*P*_3_ from isolated nuclei [46], and that we showed that the C-terminal PBR of EBP1 could bind to PtdIns(3,4,5)*P*_3_, at least among other PPIns (Figure 2D), we argued that PtdIns(3,4,5)*P*_3_ could be found in the nucleolus, in association with EBP1. Consequently, we used the PtdIns(3,4,5)*P*_3_-specific probe GRP1-PH, fused it with NLS-EGFP and examined its localization by immunofluorescence. In contrast with NLS-EGFP, which was predominantly diffuse in the nucleus, the NLS-EGFP-GRP1-PH was found in 81% of cells in the nucleus and strongly in the nucleolus together with nucleolin (Figures 4A and 4B). Furthermore, administration of the pan-PI3K inhibitor LY 294002 significantly impaired the nucleolar localization of NLS-EGFP-GRP1-PH by more than half (32%). We also examined the localization of both PtdIns(3,4,5)*P*_3_ and the nucleolar protein nucleolin by immunostaining in AU565 cells using a protocol previously established to detect detergent-resistant PPIns in the nucleus [18]. PtdIns(3,4,5)*P*_3_ was detected in the cytoplasm, nucleoplasm and nucleolus together with nucleolin (Figure 4D). The specificity of the anti-PtdIns(3,4,5)*P*_3_ antibody was validated firstly by lipid overlay assays using PIP arrays (Figure 4C). Secondly, pre-incubation of the antibody with different PPIns showed that nucleoplasmic and nucleolar staining was abolished by the presence of PtdIns(3,4,5)*P*_3_ but not by PtdIns3*P* and PtdIns(3,4)*P*_2_ (Supplementary Figure S6). Furthermore, the localization of p110β, one of the class IA phosphoinositide 3-kinase (PI3K) catalytic subunits producing PtdIns(3,4,5)*P*_3_, has previously been reported to be present in the nucleus [66]. Following immunostaining of AU565 cells, we detected p110β in the cytoplasm and strongly in the nucleolus together with NPM (Figure 4E). In summary, these results show the specificity of the anti-PtdIns(3,4,5)*P*_3_ antibody utilized, and that the nucleolar sites detected by the antibody are PtdIns(3,4,5)*P*_3_. The presence of p110β in nuclei further substantiates the existence of nucleolar PtdIns(3,4,5)*P*_3_.

### EBP1 co-localizes partially with PtdIns(3,4,5)*P*_3_ in the nucleolus

**Figure 5:**
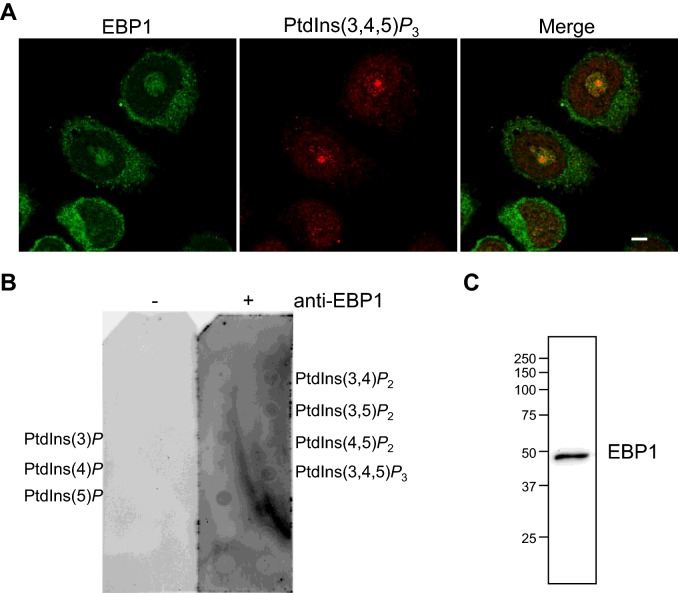
EBP1 partially co-localizes with PtdIns(3,4,5)*P*_3_ (**A**) AU565 cells co-stained with anti-EBP1 and anti-PtdIns(3,4,5)*P*3 antibodies and imaged by confocal microscopy. (**B**) PIP strips were incubated with dialysed neomycin-displaced protein extracts and protein–lipid interaction was detected with only anti-rabbit-HRP antibody (-) or with anti-EBP1 and anti-rabbit-HRP antibodies (+). (**C**) Dialysed neomycin-displaced protein extracts (20 μg) resolved by SDS/PAGE and immunoblotted with an anti-EBP1 antibody.

Considering that EBP1 and PtdIns(3,4,5)*P*_3_ were independently detected in nucleoli, we sought to determine if they co-localized by immunofluorescence. Using confocal microscopy, EBP1 was found to partially co-localize with PtdIns(3,4,5)*P*_3_ within nucleoli, suggesting therefore a potential association (Figure 5A). Binding of recombinant EBP1 to PtdIns(3,4,5)*P*_3_ is however weaker compared with binding to other PPIns (Figures 1D and 2D) and we considered that endogenous EBP1 may bind to PPIns in a different profile. To further examine the PPIn-binding properties of nuclear EBP1, we performed lipid overlay assays using neomycin-displaced protein extracts obtained from AU565 nuclei followed by detection with an anti-EBP1 antibody (Figure 5B). The pattern of interaction was similar to the recombinant protein, and in particular to the C-terminal fragment, and showed binding to most PPIns including PtdIns(3,4,5)*P*_3_. These extracts were also resolved by Western immunoblotting to demonstrate the specificity of the anti-EBP1 antibody (Figure 5C). Taken together, these results show that endogenous EBP1 and PtdIns(3,4,5)*P*_3_ can also associate in nucleoli.

### NMR analyses of EBP1–PtdIns(3,4,5)*P*_3_ interaction

**Figure 6:**
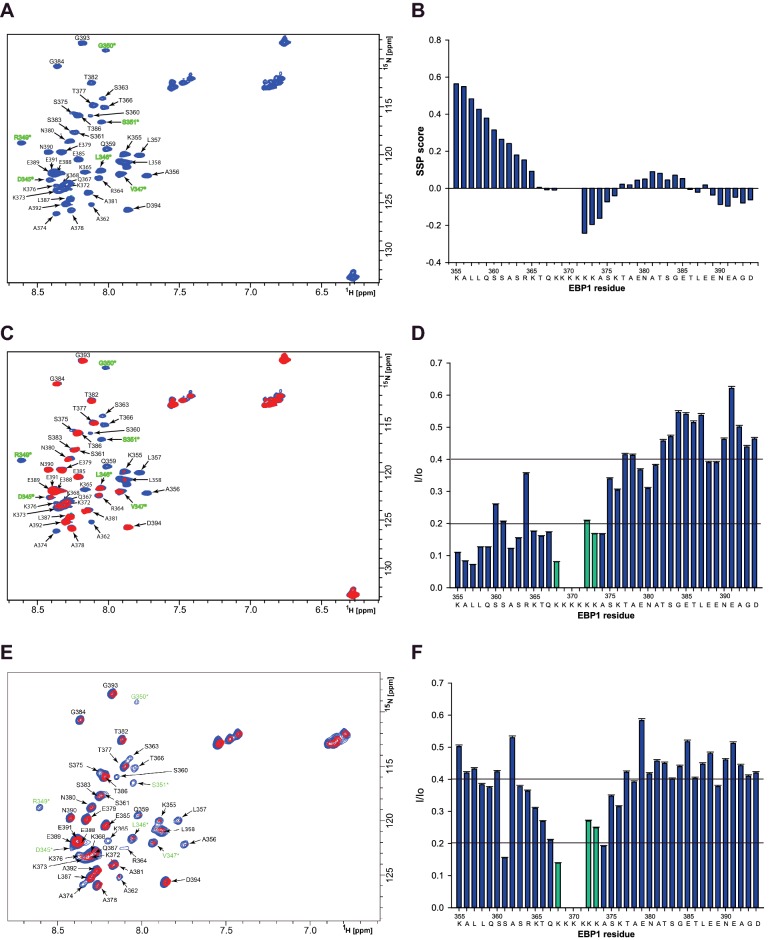
NMR characterization of the EBP1 C-terminal domain interacting with PtdIns(3,4,5)*P*_3_ (**A**) ^1^H–^15^N HSQC fingerprint spectrum of the GST-C-terminal EBP1 alone (blue). Assigned NH cross-peaks are marked in one-letter amino acid code and sequence number. Residues originating from the linker sequence positioned between the GST tag and the target sequence of EBP1 are labelled in green and marked with a star. Amino acid numbering for the linker region preceding the CTD is maintained according to the EBP1 sequence. (**B**) SSP score calculated using combined Cα and Cβ chemical shift values of the assigned EBP1 residues. Residues prone to form α-helix have a positive score, residues with a negative SSP score are prone to occupy β-sheet or extended loops. Residues in fully formed α-helixes and β-sheets are given the score of 1 and -1 respectively. (**C**) Superimposition of the ^1^H–^15^N HSQC spectra obtained for the C-terminal EBP1 in the absence (blue) and in the presence of diC16-PtdIns(3,4,5)*P*_3_ (red). (**D**) Signal intensity change upon addition of PtdIns(3,4,5)*P*_3_ calculated for EBP1 residues based on the results presented in panel C. (**E**) Superimposition of the ^1^H–^15^N HSQC spectra obtained for the C-terminal EBP1 in the absence (blue) and in the presence of diC8-PtdIns(3,4,5)*P*_3_ (red). (**F**) Signal intensity change upon addition of PtdIns(3,4,5)*P*_3_ calculated for EBP1 residues based on the results presented in panel E. Green coloured bars indicate lysines from the C-terminal PBR.

To establish further the interaction of EBP1 with PtdIns(3,4,5)*P*_3_ at the molecular level, high resolution NMR was used. The C-term PBR was examined since we have shown that it binds better to PtdIns(3,4,5)*P*_3_ in lipid overlay assays and it strongly affects the nucleolar localization of EBP1, where EBP1 and PtdIns(3,4,5)*P*_3_ co-localize. Figure 6(A) shows a typical ^1^H-^15^N HSQC fingerprint spectrum of the ^15^N isotopically labelled GST-C-terminal fragment of EBP1 (see Supplementary Figure S7 for more details). In order to first assign each amide signal to a specific residue in the protein, standard NMR experiments were performed to detect ^13^C chemical shifts and sequential connectivities. Heteronuclear 3D NMR experiments led to the chemical shift assignment of ^1^H, ^15^N and ^13^C nuclei for 37 out of 43 (86%) residues in the C-terminal fragment of EBP1. Ala^352^, Glu^353^, Leu^354^, as well as the following residues from the PBR, Lys^369^, Lys^370^, Lys^371^, were not assigned due to signal broadening beyond detection, which could be attributed to the intermediate solvent or conformational exchange rates of the protein. Altogether, the number of amide proton cross-peaks, as well as nearly complete assignment of the protein, demonstrated that the observed signals originated almost exclusively from the target protein and not from GST (see also Supplementary Figure S7). The narrow signal dispersion of the spectrum, observed for the backbone amide protons (cross-peaks positioned within 8.7–7.7 ppm range in the ^1^H dimension), indicates an overall disordered state of the protein. For proteins without a stable, folded structure, chemical shifts are essential parameters encoding local conformational propensities of the protein in solution [67,68]. To predict these propensities for the C-terminal fragment of EBP1, the obtained chemical shifts were processed to evaluate its secondary structure propensity (SSP) score using the SSP program [57]. Figure 6(B) illustrates the SSP score calculated for each assigned residue of the C-terminal fragment based on the combined Cα and Cβ chemical shifts. According to the SSP data, the C-terminal fragment has a largely disordered conformation except for the N-terminal region of the fragment (aa 355–365) showing a slight propensity to form an α-helix, consistently with the X-ray data for this region [69]. We next mapped the interaction site of the C-terminal EBP1 with either diC16- (Figure 6C) or diC8-PtdIns(3,4,5)*P*_3_ (Figure 6E). Addition of either long or short acyl chain PtdIns(3,4,5)*P*3 resulted in a decrease in signal intensity for all cross-peaks (Figures 6D and 6F), which could be due to resonance broadening caused by the presence of the lipid. Nevertheless, differences in signal ratios were observed across the C-term PBR. The most pronounced signal intensity changes were observed when diC16-PtdIns(3,4,5)*P*_3_ was added, in particular for residues located in the N-terminus part and the PBR (Lys^355^, Ala^356^, Leu^357^, Leu^358^, Gln^359^, Ala^362^, Ser^363^, Lys^365^, Thr^366^, Gln^367^, Lys^368^, as well as Lys^372^, Lys^373^, Ala^374^, Ser^375^, Lys^376^, with a ratio <0.2, and for Ser^360^, Ser^361^ and Arg^364^ with a ratio < 0.4). Similar effects were observed for the PBR residues when diC8-PtdIns(3,4,5)*P*_3_ was added, but less intensity changes were overall detected in residues in the N-terminal part. These results validate that protein–lipid contacts involve most importantly electrostatic forces between lysines and phosphates. Hydrophobic interactions between nonpolar N-terminal residues and with long acyl chains, notably in the region covered by ^356^ALL^358^, may also be involved, particularly with long hydrocarbon chains.

### A tumour-associated mutant within the C-terminal PBR affects EBP1 nucleolar localization

**Figure 7:**
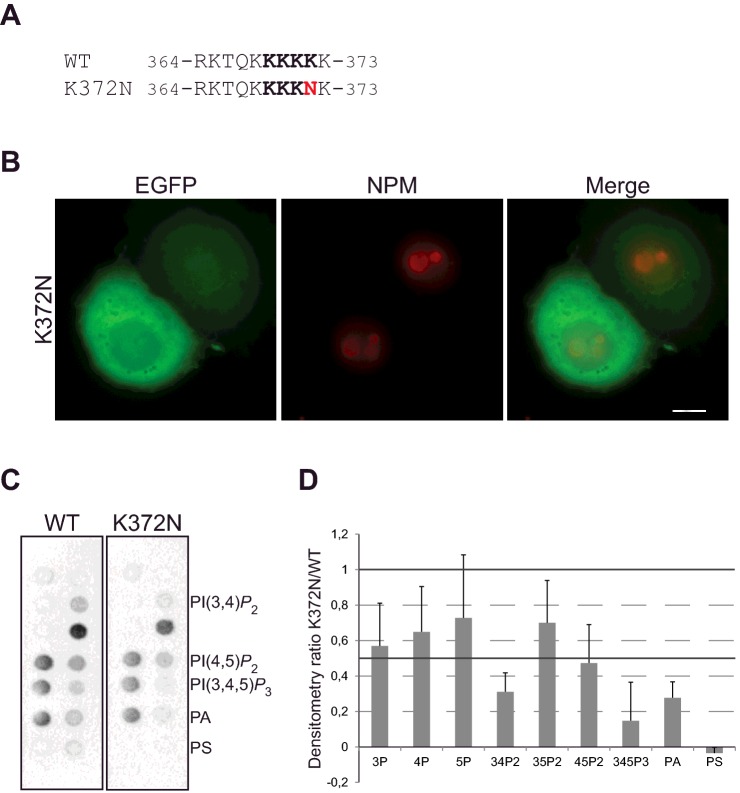
The tumour-associated EBP1 mutant K372N prevents its nucleolar localization (**A**) Alignment of the WT C-terminal K-rich motif with the K372N mutant. (**B**) AU565 cells were transfected with the EGFP-C2-EBP1 K372N mutant FL construct, stained with anti-NPM and imaged by epifluorescence microscopy (5 μmol scale bar). (**C**) PIP strips were incubated with the C-terminal domain GST-EBP1 WT and K372N proteins and protein–lipid interactions were detected using an anti-GST-HRP conjugated antibody. (**D**) Quantification of binding signal as assessed in (**C**) from three separate experiments shown as means +S.D. of densitometry ratios of K372N/WT.

EBP1 has previously been implicated to have tumour suppressor properties in different cancer types including salivary adenoid cystic and hepatocellular carcinoma (HCC), as well as prostate and breast cancer [70–73]. Consistently, the levels of EBP1 have been shown to be down-regulated in prostate cancer and HCC while its overexpression induced a decrease in cell proliferation in breast, thyroid and HCC cancer cells [70,73,74]. In contrast, recent studies have shown opposite role for EBP1 in cell proliferation since knockout mice present growth retardation [75] and overexpression induces proliferation in muscle stem cells [76]. *PA2G4*, the gene encoding EBP1, is mutated at a very low frequency (0.24% in all tumours). A mutation was however identified in the C-term PBR, i.e. K372N (Figure 7A), in an endometrial tumour sample. We therefore introduced this point mutation in EGFP-EBP FL or GST-EBP1 C-terminal constructs and examined its effect on EBP1 compartmentalization (Figure 7B) and PPIn binding (Figures 7C and 7D and Supplementary Figure S8). The K372N point mutant exhibited a more pronounced pattern #2 (cytoplasmic with either diffuse or no signal within the nucleus) in 93% of K372N-expressing cells compared with 61% in WT. Concomitantly, a decrease in pattern #1 (cytoplasmic, peri-nuclear and nucleolar) was observed with 7% in K372N-expressing cells, compared with 38% in WT. In contrast with the C-K4A mutant, this point mutation was however not sufficient to affect nuclear export as it did not lead to the retention of EBP1 in the nucleoplasm. In addition, PPIn interaction was overall only slightly reduced when the mutation was introduced in the C- terminal EBP1 construct but interestingly a stronger decrease in interaction was observed for PtdIns(3,4,5)*P*_3_, as well as for PtdIns(3,4)*P*_2_ and PA (Figures 7C and 7D). In summary, the K372N point mutant has an intermediate localization and PPIn-binding properties compared with the quadruple C-K4A mutant. The occurrence of this mutation in cancer may however point to the importance of this PBR in EBP1 function.

## DISCUSSION

We have previously reported that short motifs consisting of basic residues or PBRs following the sequence motif K/R-(*X_n_*_=3-7_)-K-X-K/R-K/R were implicated in PPIn-binding of nuclear proteins, suggesting a mode of interaction for PPIn-interacting proteins localized in the nucleus [24]. In the present study, we have identified two PBRs involved in the interaction of EBP1 with PPIns via lysine residues. The N-term PBR lies within a protruding loop although the C-term PBR is part of an unstructured sequences described by Monie et al. [77]. The two PBRs demonstrate the same profile of interaction with PPIns including the three mono-phosphorylated species and PtdIns(3,5)*P*_2_. In addition the C-terminal PBR was more promiscuous and interacted with all the other PPIns albeit more weakly. This pattern of interaction is consistent with other studies showing the importance of PBRs for PPIn binding in other nuclear proteins such as ING2 [30], Pf1 [28], SAP30L [29], and more recently UHRF1 [63] and TAF3 [42]. In particular, mono-phosphorylated PPIns interact with SAP30L, Pf1, TAF3 and ING2 whereas PtdIns(3,5)*P*_2_ interacts with Pf1 and TAF3. The consistency of the pattern of interaction of these PBRs with certain PPIns is rather interesting and may hence point to specificity due to the position of the phosphate on the inositol ring for short basic motifs. Clusters of basic residues in PBRs may hence preferentially accommodate interaction with either one phosphate or PtdIns(3,5)*P*_2_ with sufficiently spaced phosphates. Interestingly, SAP30L binds to mono-phosphorylated PPIns via a stretch of three basic residues within its PBR which also functions as an NLS adjacent to its DNA binding domain. The C-terminal PBR of EBP1, which contains six adjacent lysines, binds more extensively to PPIns and includes in addition the other bis-phosphorylated as well as PtdIns(3,4,5)*P*_3_. The higher number of lysines in this motif may hence offer more binding probabilities and alternatives to several PPIns with differently spaced phosphates on the inositol ring, at least *in vitro*.

The two PBRs may act independently and may provide different cellular functions to EBP1. Considering that PPIns are known to target proteins to different sub-cellular localizations, the two PBRs may regulate the different cellular locations of EBP1 allowing for potential roles in the nucleolus and cytoplasm bound to ribosomes [78]. We showed that the mutated N-terminal PBR restricted EBP1 mostly to the cytoplasm and exhibited either little or a diffuse nuclear signal in the majority of cells. The N-terminal PBR may hence cooperate with the first 48 amino acids, including the two lysines K20 and K22, shown previously to be necessary for the nuclear localization of EBP1 [53]. The C-terminal PBR is clearly important for the nucleolar localization of EBP1, and the sequestration of the C-terminal PBR mutant in the nucleoplasm coincident with its nucleolar exclusion suggest that this PBR also plays a role in nuclear export of EBP1. Considering that the first 48 amino acids in EBP1 are critical for nuclear targeting [53], the C-terminal PBR mutant is therefore able to reach the nucleus but is however unable to be retained in the nucleolus. Another nuclear protein, SAP30L, was shown to harbour a NoLS, located C-terminal of its NLS, and to contribute to PPIn interaction in addition to the NLS/PBR described previously [29]. The NoLS in SAP30L consists of the following polybasic sequence _120_RRYKRHYK_127_ and when all these residues were mutated to alanines, SAP30L was excluded from the nucleolus but retained in the nucleus [79], which is consistent with the behaviour of the C-terminal PBR of EBP1. These studies raise the possibility of multi functions for NoLS beyond nucleolar targeting, including nuclear export as well as PPIn interaction.

The importance of the C-term PBR in the localization of EBP1 is highlighted by the occurrence of a mutation in endometrial cancer, K372N, which we have demonstrated to be important in both PPIn interaction and nucleolar localization. The importance of EBP1 compartmentalization in cancer takes precedence in salivary cancer in which EBP1 is sequestered in the cytoplasm in tumour areas whereas adjacent normal cells localize EBP1 in both the cytoplasm and nucleus [80]. Sub-nuclear details could however not be identified in the present study to distinguish nucleolar staining. The sub-cellular localization of EBP1 has moreover been suggested to be important for its function and its nucleolar localization correlates with its role in cell proliferation suppression [53]. Based on this, we also suggest that the C-terminal PBR is involved in regulating the cellular function of EBP1 by inducing changes in its sub-cellular localization. Cancer cells may hence benefit from targeting the C-terminal motif to alter the sub-cellular localization of EBP1 and perhaps more specifically by preventing EBP1 from entering the nucleolus. The role of EBP1 in the nucleolus remains to be identified.

The molecular mode of retention of EBP1 in the nucleolus is not clear but it is tempting to suggest that PtdIns(3,4,5)*P*_3_ interaction could play a role. Firstly, EBP1 association with PtdIns(3,4,5)*P*_3_ was demonstrated in several ways: (1) the C-term PBR binds to this PPIn species *in vitro*, as shown by lipid overlay assays and NMR analyses, (2) endogenous nuclear EBP1 has binding capacity as shown by lipid overlay assays. Secondly, partial co-localization of endogenous EBP1 with PtdIns(3,4,5)*P*_3_ within the nucleolus was observed, and this could be consistent with a functional association. In addition, NMR studies revealed that other residues located N-terminal of the PBR, including the nonpolar ^356^ALL^358^ motif, made contact with PtdIns(3,4,5)*P*_3_ containing 16-carbon but not 8-carbon acyl chains. This would suggest that the acyl chains contribute to the interaction together with the head group. The participation of acyl chains in protein–PPIn interaction is a mechanism that has previously been suggested in several nuclear proteins identified to bind diC16-PtdIns(3,4,5)*P*_3_ without the involvement of structured PI-binding domain [27]. Thus, the identification of nuclear proteins which can potentially accommodate the acyl chains of PPIns may provide an explanation for the presence of PPIns in the non-membranous environment of the nucleus and notably the nucleolus.

The presence of PtdIns(3,4,5)*P*_3_ in the nucleus has been reported previously [16] and in particular in cells treated with γ-irradiation [81] but the presence of this PPIn and the PI3K catalytic subunit p110β in the nucleolus is reported for the first time in the present study. A minor pool of nuclear PtdIns(4,5)*P*_2_ has previously been detected in the nucleolus of asynchronously growing cells [18,82–84], which could hence be the source of PtdIns(3,4,5)*P*_3_ production in this nuclear site. We have initially identified EBP1 as a potential PtdIns(4,5)*P*_2_-binding protein by pull down assay and mass spectrometry [24] and we have shown in the present study that the C-terminal PBR of EBP1 can bind directly to both PtdIns(4,5)*P*_2_ and PtdIns(3,4,5)*P*_3_, the two PPIns found to be present in the nucleolus. EBP1 may therefore associate with PtdIns(4,5)*P*_2_ as well as PtdIns(3,4,5)*P*_3_ in this nuclear site. We observed that the pattern of detection of nucleolar PtdIns(3,4,5)*P*_3_ varies in asynchronous AU565 (Figure 4C) and HeLa (results not shown) cells and can consist of either a punctate and intense signal within nucleoli or a more diffuse distribution in both the nucleoplasm and nucleolus. We suggest therefore that EBP1 may associate differentially with PtdIns(3,4,5)*P*_3_ or PtdIns(4,5)*P*_2_ under certain conditions of the cell cycle. Consistently p110β has been previously reported to be active during G1 to S phase transition of the cell cycle [85]. We suggest therefore that changes in p110β activity could explain the differential pattern of PtdIns(3,4,5)*P*_3_ in the nucleolus and nucleoplasm and regulate the function of EBP1 and we are currently pursuing this line of study.

Although the localization of EBP1 in the nucleolus can be due to the interaction of its C-terminal PBR with PtdIns(3,4,5)*P*_3_, another mode of retention of EBP1 is possible via rRNA-interaction. The C-terminal region of EBP1 spanning amino acids 361–394 was shown to be necessary for RNA interaction [77], including the processed rRNA species 18S, 28S and 5.8S [53]. This region comprises the C-terminal PBR described in the present study and the lysines present within the PBR shown to be involved in both nucleolar retention and PPIn interaction could be responsible for RNA binding via electrostatic interactions. Considering that this PBR is responsible for both PPIn interaction and nucleolar retention and is implicated in nucleic acid binding, we would suggest that PtdIns(3,4,5)*P*_3_ and rRNA compete for binding to the C-terminal PBR. This scenario has indeed been reported for the HIV-1 viral protein Gag where binding of RNA to a highly basic region was demonstrated as a mechanism to prevent PtdIns(4,5)*P*_2_-mediated binding to the plasma membrane [86]. In addition, PtdIns(3,4,5)*P*_3_ interacts with NPM via lysine residues [46], which are part of a basic, intrinsic disordered region involved in RNA interaction [87]. Finally, PtdIns(3,4,5)*P*_3_ binds ALY, a protein regulating mRNA export via basic residues [35], and this interaction contributes to ALY-mediated recognition of specific mRNA transcripts for their nuclear export [37]. In light of our results obtained with the C-term PBR of EBP1 and studies in NPM and ALY, it is tempting to suggest that PtdIns(3,4,5)*P*_3_ may regulate protein–RNA interaction utilizing basic motifs in disordered regions which have dual functions in PPIn and RNA interaction.

In conclusion, we have shown that the PBRs identified in EBP1 have a dual function as they contribute to PPIn interaction, potentially via electrostatic interactions, as well as nucleolar localization. Considering that sub-cellular localization often correlates with function, PBRs may provide a molecular mechanism allowing EBP1 to switch between different sub-cellular compartments and functions due to their interaction with PPIns. In addition, this is the first report providing evidence of the presence of PtdIns(3,4,5)*P*_3_ as well as the class I PI3K catalytic subunit p110β in the nucleolus. EBP1 binds PtdIns(3,4,5)*P*_3_ and this association is detected in the nucleolus. Our data imply novel regulation of nucleolar functions by PtdIns(3,4,5)*P*_3_, which are lines of research that we are currently pursuing.

## Abbreviations

BASP1: brain acid soluble protein 1
EBP1: ErbB3 binding protein 1
FL: full length
ING2: inhibitor of growth protein 2
LRH-1: liver receptor homologue-1
NPM: nucleophosmin
PA2G4: proliferation-associated protein 2G4
PBR: polybasic region
Pf1: plant homeodomain zinc finger 1
PI3K: phosphoinositide 3-kinase
PPIn: polyphosphoinositide
PtdIns: phosphatidylinositol
SAP30L: Sin3A-associated protein 30 like
SF-1: steroidogenic factor-1
SSP: secondary structure propensity
TAF3: TATA box binding protein-associated factor 3
